# Supplementary figures and images for: Population level differences in overwintering survivorship of blue crabs (Callinectes sapidus): A caution on extrapolating climate sensitivities along latitudinal gradients
Source: PLoS One. 2021 Sep 21;16(9):e0257569. doi: 10.1371/journal.pone.0257569 (PMC8454986; doi:10.1371/journal.pone.0257569)

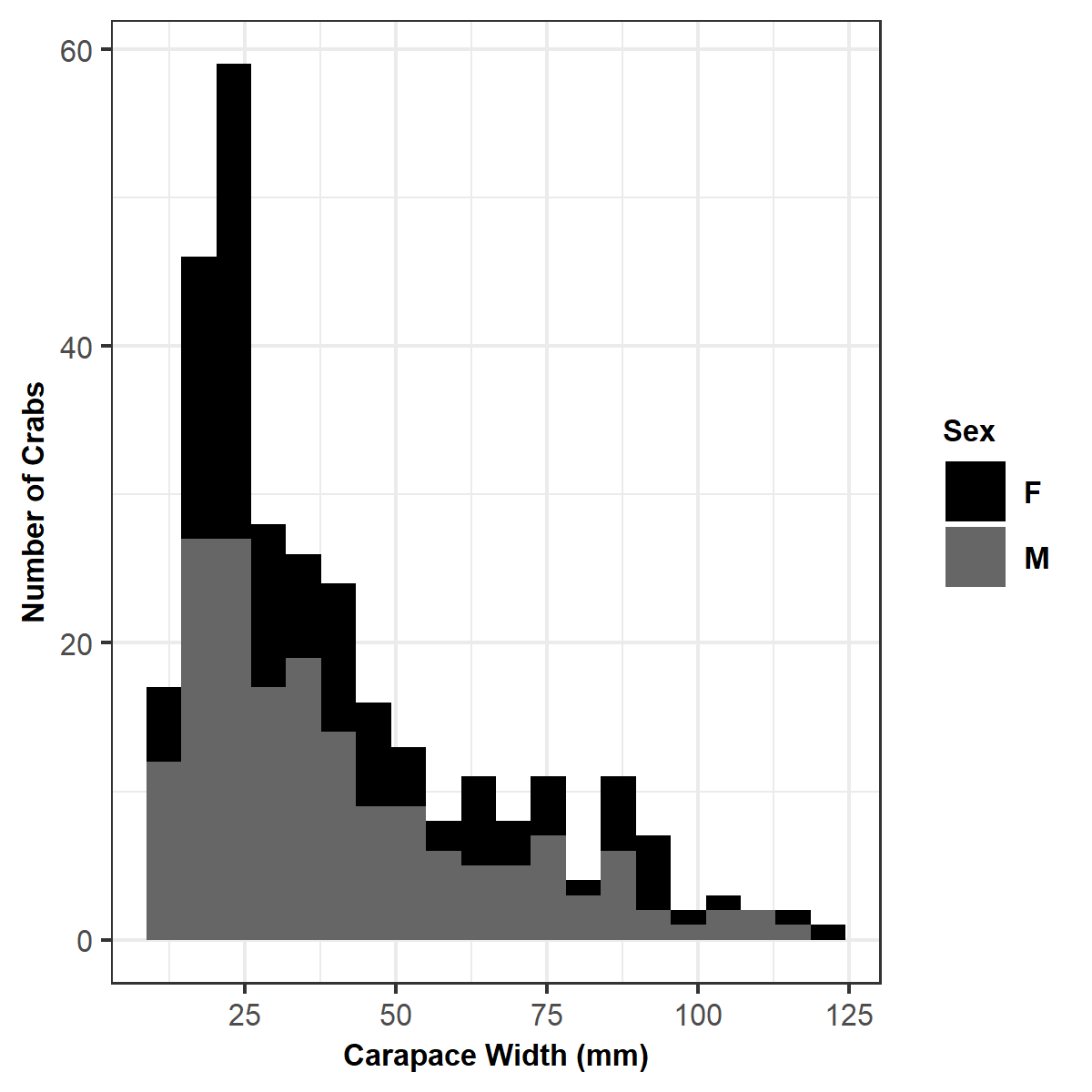

Supplement: S1 Fig — Gray bars show the number of male crabs used in all experiments in all years in 10 mm size bins. Black bars show the number of females. (TIFF) [file pone.0257569.s001.tiff]
