# Supplementary material for: Population level differences in overwintering survivorship of blue crabs (Callinectes sapidus): A caution on extrapolating climate sensitivities along latitudinal gradients
Source: PLoS One. 2021 Sep 21;16(9):e0257569. doi: 10.1371/journal.pone.0257569 (PMC8454986; doi:10.1371/journal.pone.0257569)
Supplement: S2 Table — (DOCX) [file pone.0257569.s003.docx]

| **Distribution** | **Covariates** | **df** | **AIC** | **dAIC** | **weight** |
| --- | --- | --- | --- | --- | --- |
| Gen Gamma | T, S, CW, T*CW | 7 | 1830.8 | 0 | 0.4361 |
| Gen Gamma | T, S, CW, T*S, T*CW | 8 | 1831.9 | 1.1 | 0.2509 |
| Weibull | T, S, CW, T*CW | 6 | 1834.2 | 3.5 | 0.0773 |
| Exponential | T, S, CW, T*CW | 5 | 1835 | 4.3 | 0.0512 |
| Weibull | T, S, CW, T*S, T*CW | 7 | 1835.2 | 4.4 | 0.0482 |
| Weibull | T, S, CW, T*CW, S*CW | 7 | 1836 | 5.3 | 0.0315 |
| Exponential | T, S, CW, T*S, T*CW | 6 | 1836.4 | 5.6 | 0.0264 |
| Exponential | T, S, CW, T*CW, S*CW | 6 | 1836.9 | 6.1 | 0.0205 |
| Weibull | T, S, CW, T*S, T*CW, S*CW | 8 | 1837.1 | 6.3 | 0.0183 |
| Weibull | T, S, CW, T*S, T*CW, S*CW, T*CW*S | 9 | 1838.2 | 7.4 | 0.0108 |
| Exponential | T, S, CW, T*S, T*CW, S*CW | 7 | 1838.3 | 7.5 | 0.01 |
| Gen Gamma | T, S | 5 | 1839.4 | 8.6 | 0.0059 |
| Exponential | T, S, CW, T*S, T*CW, S*CW, T*CW*S | 8 | 1839.6 | 8.8 | 0.0053 |
| Gen Gamma | T*S | 6 | 1841.2 | 10.4 | 0.0024 |
| Gen Gamma | T, S, CW | 6 | 1841.2 | 10.5 | 0.0023 |
| Gen Gamma | T, S, CW, T*S | 7 | 1843 | 12.3 | <0.001 |
| Exponential | T, S | 3 | 1844.4 | 13.6 | <0.001 |
| Weibull | T, S | 4 | 1844.5 | 13.7 | <0.001 |
| Weibull | T*S | 5 | 1845.9 | 15.2 | <0.001 |
| Exponential | T*S | 4 | 1846 | 15.3 | <0.001 |
| Exponential | T, S, CW | 4 | 1846.3 | 15.6 | <0.001 |
| Weibull | T, S, CW | 5 | 1846.5 | 15.7 | <0.001 |
| Weibull | T, S, CW, T*S | 6 | 1847.9 | 17.1 | <0.001 |
| Exponential | T, S, CW, T*S | 5 | 1847.9 | 17.2 | <0.001 |
| Exponential | T, S, CW, S*CW | 5 | 1848.3 | 17.5 | <0.001 |
| Weibull | T, S, CW, S*CW | 6 | 1848.4 | 17.7 | <0.001 |
| Weibull | T, S, CW, T*S, S*CW | 7 | 1849.8 | 19 | <0.001 |
| Exponential | T, S, CW, T*S, S*CW | 6 | 1849.8 | 19.1 | <0.001 |
| Gen Gamma | S, CW | 5 | 1863.1 | 32.4 | <0.001 |
| Gen Gamma | S | 4 | 1863.9 | 33.1 | <0.001 |
| Gen Gamma | S | 2 | 1864 | 33.3 | <0.001 |
| Exponential | S, CW | 3 | 1864.8 | 34.1 | <0.001 |
| Weibull | S | 3 | 1865.8 | 35.1 | <0.001 |
| Weibull | S, CW | 4 | 1866.5 | 35.8 | <0.001 |
| Exponential | S*CW | 4 | 1866.8 | 36 | <0.001 |
| Weibull | T, S, CW, S*CW | 5 | 1868.4 | 37.7 | <0.001 |
| Lognormal | T, S, CW, T*CW | 6 | 1874 | 43.2 | <0.001 |
| Lognormal | T, S, CW, T*S, T*CW | 7 | 1875.5 | 44.8 | <0.001 |
| Lognormal | T, S, CW, T*CW, S*CW | 7 | 1875.6 | 44.8 | <0.001 |
| Lognormal | T, S, CW, T*S, T*CW, S*CW | 8 | 1877.2 | 46.4 | <0.001 |
| Gen Gamma | T, S, CW, T*CW, S*CW | 8 | 1877.8 | 47 | <0.001 |
| Lognormal | T, S | 4 | 1882.9 | 52.2 | <0.001 |
| Lognormal | T*S | 5 | 1884.4 | 53.6 | <0.001 |
| Lognormal | T, S, CW | 5 | 1884.9 | 54.2 | <0.001 |
| Lognormal | T, S, CW, S*CW | 6 | 1886 | 55.3 | <0.001 |
| Lognormal | T, S, CW, T*S | 6 | 1886.3 | 55.6 | <0.001 |
| Lognormal | T, S, CW, T*S, S*CW | 7 | 1887.5 | 56.8 | <0.001 |
| Lognormal | S | 3 | 1894 | 63.3 | <0.001 |
| Lognormal | S, CW | 4 | 1896 | 65.3 | <0.001 |
| Lognormal | S*CW | 5 | 1897.5 | 66.7 | <0.001 |
| Exponential | T, S, CW, T*CW | 4 | 1913.7 | 82.9 | <0.001 |
| Weibull | T, S, CW, T*CW | 5 | 1915.5 | 84.7 | <0.001 |
| Exponential | T | 2 | 1916.2 | 85.4 | <0.001 |
| Gen Gamma | T, S, CW, T*CW | 6 | 1917.2 | 86.4 | <0.001 |
| Weibull | T | 3 | 1917.9 | 87.2 | <0.001 |
| Exponential | T, CW | 3 | 1918.2 | 87.4 | <0.001 |
| Gen Gamma | T | 4 | 1918.3 | 87.6 | <0.001 |
| Weibull | T, CW | 4 | 1919.9 | 89.2 | <0.001 |
| Gen Gamma | T, CW | 5 | 1920.3 | 89.6 | <0.001 |
| Lognormal | T*CW | 5 | 1930.6 | 99.8 | <0.001 |
| Lognormal | T | 3 | 1935.4 | 104.6 | <0.001 |
| Exponential | CW | 2 | 1936.5 | 105.7 | <0.001 |
| Gen Gamma | CW | 4 | 1937 | 106.2 | <0.001 |
| Weibull | CW | 3 | 1937.4 | 106.6 | <0.001 |
| Lognormal | T, CW | 4 | 1937.4 | 106.6 | <0.001 |
| Lognormal | CW | 3 | 1951.5 | 120.8 | <0.001 |
