# Supplementary material for: Population level differences in overwintering survivorship of blue crabs (Callinectes sapidus): A caution on extrapolating climate sensitivities along latitudinal gradients
Source: PLoS One. 2021 Sep 21;16(9):e0257569. doi: 10.1371/journal.pone.0257569 (PMC8454986; doi:10.1371/journal.pone.0257569)
Supplement: S3 Table — (DOCX) [file pone.0257569.s004.docx]

| **Distribution** | **Covariates** | **df** | **AIC** | **dAIC** | **weight** |
| --- | --- | --- | --- | --- | --- |
| Exponential | T, S, CW, T*S, T*CW | 6 | 3030.1 | 0 | 0.1973 |
| Weibull | T, S, CW, T*S, T*CW | 7 | 3030.3 | 0.2 | 0.178 |
| Gen Gamma | T, S, CW, T*S, T*CW | 8 | 3031.5 | 1.3 | 0.1011 |
| Exponential | T, S, CW, T*CW | 5 | 3032 | 1.9 | 0.0767 |
| Exponential | T, S, CW, T*S, T*CW, S*CW | 7 | 3032.1 | 2 | 0.0726 |
| Weibull | T, S, CW, T*S, T*CW, S*CW | 8 | 3032.3 | 2.2 | 0.0655 |
| Weibull | T, S, CW, T*CW | 6 | 3032.6 | 2.4 | 0.0584 |
| Gen Gamma | T, S, CW, T*CW | 7 | 3032.6 | 2.5 | 0.0568 |
| Exponential | T, S, CW, T*CW, S*CW | 6 | 3033.9 | 3.8 | 0.0299 |
| Exponential | T, S, CW, T*S, T*CW, S*CW, TCWS | 8 | 3034 | 3.9 | 0.0284 |
| Weibull | T, S, CW, T*S, T*CW, S*CW, T*CW*S | 9 | 3034.2 | 4.1 | 0.026 |
| Weibull | T, S, CW, T*CW, S*CW | 7 | 3034.4 | 4.3 | 0.0229 |
| Gen Gamma | T,S | 5 | 3035.7 | 5.5 | 0.0124 |
| Gen Gamma | T*S | 6 | 3035.9 | 5.8 | 0.0109 |
| Gen Gamma | T*S | 4 | 3036.1 | 6 | 0.01 |
| Exponential | T,S | 3 | 3036.7 | 6.6 | 0.0074 |
| Weibull | T*S | 5 | 3036.7 | 6.6 | 0.0074 |
| Exponential | T, S, CW, T*S | 5 | 3037.2 | 7.1 | 0.0057 |
| Gen Gamma | T,S,CW | 6 | 3037.3 | 7.2 | 0.0055 |
| Gen Gamma | T, S, CW, T*S | 7 | 3037.5 | 7.4 | 0.0049 |
| Weibull | T,S | 4 | 3037.5 | 7.4 | 0.0049 |
| Weibull | T, S, CW, T*S | 6 | 3037.8 | 7.6 | 0.0043 |
| Exponential | T, S, CW | 4 | 3037.9 | 7.8 | 0.004 |
| Weibull | T, S, CW | 5 | 3038.7 | 8.6 | 0.0027 |
| Exponential | T, S, CW, T*S, S*CW | 6 | 3039.2 | 9.1 | 0.0021 |
| Weibull | T, S, CW, T*S, S*CW | 7 | 3039.8 | 9.6 | 0.0016 |
| Exponential | T, S, CW, S*CW | 5 | 3039.9 | 9.8 | 0.0015 |
| Weibull | T, S, CW, S*CW | 6 | 3040.7 | 10.6 | <0.001 |
| Gen Gamma | S | 4 | 3051.8 | 21.6 | <0.001 |
| Exponential | S | 2 | 3052 | 21.8 | <0.001 |
| Weibull | S | 3 | 3053.3 | 23.2 | <0.001 |
| Gen Gamma | S, CW | 5 | 3053.6 | 23.5 | <0.001 |
| Exponential | S, CW | 3 | 3054 | 23.8 | <0.001 |
| Weibull | S, CW | 4 | 3055.3 | 25.2 | <0.001 |
| Exponential | S*CW | 4 | 3055.9 | 25.7 | <0.001 |
| Weibull | S*CW | 5 | 3057.2 | 27.1 | <0.001 |
| Lognormal | T, S, CW, T*S, T*CW | 7 | 3067.8 | 37.6 | <0.001 |
| Lognormal | T, S, CW, T*S, T*CW, S*CW | 8 | 3069.6 | 39.4 | <0.001 |
| Lognormal | T, S, CW, T*CW | 6 | 3069.8 | 39.7 | <0.001 |
| Lognormal | T, S, CW, T*CW, S*CW | 7 | 3071.4 | 41.3 | <0.001 |
| Gen Gamma | T | 4 | 3071.7 | 41.6 | <0.001 |
| Gen Gamma | T*CW | 6 | 3072.5 | 42.3 | <0.001 |
| Exponential | T*CW | 4 | 3073.7 | 43.5 | <0.001 |
| Gen Gamma | T,CW | 5 | 3073.7 | 43.6 | <0.001 |
| Gen Gamma | T, S, CW, T*CW, S*CW | 8 | 3074.4 | 44.3 | <0.001 |
| Weibull | T*CW | 5 | 3074.9 | 44.8 | <0.001 |
| Lognormal | T*S | 5 | 3075.1 | 44.9 | <0.001 |
| Lognormal | T, S, CW, T*S | 6 | 3075.1 | 45 | <0.001 |
| Exponential | T | 2 | 3075.2 | 45.1 | <0.001 |
| Lognormal | T, S, CW, T*S, S*CW | 7 | 3076.6 | 46.5 | <0.001 |
| Weibull | T | 3 | 3076.6 | 46.5 | <0.001 |
| Lognormal | T,S,CW | 4 | 3076.7 | 46.5 | <0.001 |
| Lognormal | T,S,CW | 5 | 3076.9 | 46.7 | <0.001 |
| Exponential | T,CW | 3 | 3077.1 | 46.9 | <0.001 |
| Lognormal | T, S, CW, S*CW | 6 | 3078.2 | 48 | <0.001 |
| Weibull | T,CW | 4 | 3078.4 | 48.3 | <0.001 |
| Lognormal | S | 3 | 3087 | 56.9 | <0.001 |
| Lognormal | S,CW | 4 | 3087.7 | 57.6 | <0.001 |
| Gen Gamma | CW | 4 | 3088.6 | 58.4 | <0.001 |
| Lognormal | S*CW | 5 | 3089.2 | 59.1 | <0.001 |
| Exponential | CW | 2 | 3092.7 | 62.6 | <0.001 |
| Weibull | CW | 3 | 3094.4 | 64.3 | <0.001 |
| Lognormal | T*CW | 5 | 3106.3 | 76.1 | <0.001 |
| Lognormal | T | 3 | 3110.9 | 80.7 | <0.001 |
| Lognormal | T, CW | 4 | 3111.8 | 81.7 | <0.001 |
| Lognormal | CW | 3 | 3125.4 | 95.3 | <0.001 |
