# Supplementary material for: Population level differences in overwintering survivorship of blue crabs (Callinectes sapidus): A caution on extrapolating climate sensitivities along latitudinal gradients
Source: PLoS One. 2021 Sep 21;16(9):e0257569. doi: 10.1371/journal.pone.0257569 (PMC8454986; doi:10.1371/journal.pone.0257569)
Supplement: Attachment — Submitted filename: Response to Reviewers.docx [file pone.0257569.s005.docx]

Dear Mr. Charles William Martin,

Thank you for giving us the opportunity to submit a revised draft of the manuscript titled “Population level differences in overwintering survivorship of blue crabs (Callinectes sapidus): a caution on extrapolating climate sensitivities along latitudinal gradients” to *PLOS ONE*. We appreciate the time and effort that you and the reviewers have dedicated to providing your valuable feedback on our manuscript. We are grateful to the reviewers for their insightful comments. We have been able to incorporate changes to reflect most of the suggestions provided by the reviewers and have highlighted the changes in the copy with track changes.

Here is a point-by-point response to the reviewers’ comments and concerns.

**Journal Requirements**

1. We have double checked that all journal formatting requirements have been met, including those for file naming.
2. We have included an additional supplemental table with all the GPS coordinates for Great South Bay trawl collections, and we also provided the coordinates of Chesapeake Bay seining in the text.
3. We did obtain permission to share the full dataset including the data from (Bauer & Miller, 2010a)**.** It can be found at FigShare at the following DOI: [10.6084/m9.figshare.13990961](https://doi.org/10.6084/m9.figshare.13990961)

**General Comments from Reviewer 1**

**Comment 1**: *I understand that each crab was in its own container in all of the experiments, but given that multiple crabs were sharing a water bath, I wonder if there is some lack of independence here. Should “water bath ID” be included in the models in some way? I’d like some more information from the authors on the independence of the crabs in a given bath. I am not particularly familiar with the statistical methods used by the authors, however, so their analyses may be completely correct.*

**Response**: Having separate tanks as the experimental unit within a water bath is common practice for aquatic and marine experiments because it is logistically impossible to have a chilling/heating unit for each tank. We believe it is akin to having multiple cages within one control temperature room for experiments on terrestrial organisms where the temperature is controlled by one unit. The water temperature is fairly homogeneous for the entire experiment, but water flow is slightly different in each tank and crabs do not interact with each other at all in their separate compartments. Also, since we are not doing an ANOVA, “block effects” are difficult to assess, but in years 2 and 3 we tried to assess if there was a something akin to a block effect. In the first year, there was only one water bath per treatment so there was no reason to assess the effect of water bath. In the second year we had 3 water baths per treatment, and in the third year we had 2 water baths per treatment. The “water bath ID” suggested by Reviewer 1 is equivalent to grouping crabs by water bath. To assess a possible grouping effect, we performed a log-rank test for each treatment in year 2 and 3. The log-rank test assessed the equality of survival curves for the groups. In year 3, water bath was not significant (p = 0.989). In year 2 for just the most severe treatment, the water bath effect was significant (p= 0.03); however, the crabs for this treatment happened to be larger than in other treatments. The water bath effect became nonsignificant when we stratified by crab size (p= 0.3), and we included “size” as a variable in our model.

**Comment 2**: *It is unclear if all three experiments were lumped together for statistical analyses, or if each was analyzed separately. It seems to me that they should be analyzed separately since the experimental design was different for each. If analyzed together (which may be possible), it needs to be clearly discussed why the authors feel this is the best approach.*

**Response**: While we did initially analyze the data from each experimental year separately, it was always our intention to develop a robust model that was based on a wide range of input temperatures and salinities as recommended by Bauer and Miller. For this reason, we deliberately combined the data in the analysis because we tested different temperatures in each year. While it would have been ideal to have been able to test all the desired temperatures and salinities in one year, that would not have been feasible with our resources and facilities. Furthermore, the top models that resulted from the individual year analysis included the same parameters as the models from the data that was combined across years.

**Comment 3**: *The results were difficult to review as the figure captions were within the results text. It was difficult to tell what was a caption and what was meant to be text in the results section.*

**Response:** The journal formatting requires figure captions below the first paragraph in which they are mentioned. We have added some extra space to make it clearer.

**Line Comments from Reviewer 1**

**Line 36**: *Not clear what you mean by “bay”. Perhaps reword to “collection site” or “estuary of origin”. Same elsewhere in the abstract.*

**Response**: We changed it to estuary of origin in both occurrences in the abstract

**Line 46**: *I wouldn’t call Chesapeake the center of the historic distribution of the blue crab, considering their native range extends to South America.*

**Response**: Agreed, we removed the term “center of the historic distribution” and edited the text to make it a bit more general.

**Line 68**: *“However, this necessary assumption is rarely tested”- I would argue that among-population variation in thermal tolerance, thermal sensitivity, and thermal performance breadth have been tested quite heavily. See Angilletta, M. J. (2009). Thermal adaptation: a theoretical and empirical synthesis. Oxford University Press. You are correct that these measures of thermal tolerance and sensitivity are rarely constant across populations, but this is not something that has rarely been tested. There is quite a lot of theoretical and empirical work on this subject.*

**Response**: It seems that this assumption is much better understood in terrestrial organisms, so we changed it from rarely to not always explicitly tested or accounted for in marine organisms.

**Line 172-173**: *Better to word as “Rome et al. (2005) observed significantly higher experimental mortality rates…” since Rome is a person, and you’re not talking about experimental mortality rates in a person (which is what the current wording suggests).*
**Response:** Changed, thank you for the suggestion.

**Line 182**: *Remove comma after “Although”*

**Response:** Done

**Line 184:** *I suggest adding additional references for no existence of distinct genetic populations. There was been a lot of work done in this area since McMillen-Jackson and Bert published their study in 2004.*

**Response:** Thank you for the suggestion. We perused this extensive literature and incorporated it into the section that discusses diversity in the discussion (line 496)

**Line 227-228:** *I agree they don’t grow, but do they also not eat below this temperature?*

**Response:** It appears that they do not eat below this temperature. While this has not been explicitly tested or confirmed in field observations, Bauer and Miller (2010a) observed that crab feeding in the lab decreased as the temperature dropped below 10°C and ceased shortly thereafter. Furthermore, Brylawski and Miller (2003) set 10°C as the zero consumption point in their bioenergetic model of blue crabs.

**Line 256***: “a sea tables” should this be “a sea table”*

**Response:** Edited

**Line 315-316:** *I question the classification of >60 mm as adults. I realize this is just a name for the size group, but very few 60 mm crabs are mature. Some are, of course, and perhaps more now than 10 years ago, but size at 50% maturity is typically closer to 100 mm or even higher than that.*

**Response:** We had certainly not intended these terms to indicate maturity; as you pointed out, they are just names for the size groups. To prevent confusion, we have decided to just use small, medium, and large. We edited this throughout the text and in figures.

**Line 317**: *I suggest changing wording to “Mortality rates varied among size classes”.*

**Response:** That is much better, changed

**Line** 323: *Instead of “the effect of bay was…” I suggest “Without considering size, mortality rates differed significantly between bays”.*

**Response:** We agree, edited as per your suggestion.

**Line** 508-510: *I’m not familiar with the fish literature, but I’m not sure how this statement is relevant for blue crabs. Blue crabs show faster growth (measured as size increase per unit time) in more southern populations. If this applies for blue crabs and other crustaceans, please reference accordingly.*

**Response:** Thank you for this comment. There was too much fish literature in that section, so we have removed those and edited the text to include more references for crustacean latitudinal growth patterns. Although, it should be noted that the literature on thermal tolerance in marine systems is dominated by fishes.

**Line 516-517**. *The temperature-size rule has also been supported by studies on juvenile blue crabs, for example: Cunningham, S. R., & Darnell, M. Z. (2015). Temperature-dependent growth and molting in early juvenile blue crabs Callinectes sapidus. Journal of Shellfish Research, 34(2), 505-510. That paper examined growth rates in juvenile blue crabs and found growth patterns that would be expected based on the TSR, although growth was not followed all the way to maturity. This study found decreased intermolt period and decreased growth per molt (but higher absolute growth rates) at higher temperatures, leading to decreased size at each instar at higher temperatures.*

**Response:** As per my response to the comment above, this entire section has been significantly reworked to focus on crustacean and blue crab growth. We included the paper you suggested and a few other new references to better explain what has been shown for blue crabs and the temperature size rule.

**Line 534**: *Might be worth reminding the reader that the higher winter mortality rates were observed under the same winter temperature.*

**Response:** That certainly strengthens the sentence. Edited although this sentence has now been moved to a different section of the discussion.

**General Comments from Reviewer 2**

**Comment 1:** *In general, the manuscript is organized effectively, the experiments and analytical methods are appropriate and clearly described, and the main conclusions well supported by the results. A few additional details about experimental animals would be helpful, and some sections of the discussion could be reduced or eliminated to avoid straying too far beyond the results of the study.*

**Response**: We added some additional information about the animal collection and transportation. We also made some changes and cuts to the discussion section.

**Line Comments from Reviewer 2**

**Introductory information:** *Was a permit required for Chesapeake Bay collections? Could you include the link to the data on figshare?*

**Response**: Crabs from the Virginia portion of Chesapeake Bay were collected with the help of colleagues at VIMS under VA law 28.2-1101.

<https://law.lis.virginia.gov/vacode/title28.2/chapter11/section28.2-1101/>

We did obtain permission to share the full dataset including the data from (Bauer & Miller, 2010a)**.** It can be found at FigShare at the following DOI: [10.6084/m9.figshare.13990961](https://doi.org/10.6084/m9.figshare.13990961)

**Line 36**: *define “bay” or choose a different term that is more apparent (e.g. source location).*

**Response**: We used estuary of origin as per reviewer 1’s comment

**Line 44**: *I’m not sure this statement is conceptually sound. I think the authors mean that warming could confer a benefit to Great South Bay crabs but this would primarily be by shifting the range edge further north so that this location may no longer be at the range edge. There’s no evidence in the study that crabs at the range edge (perhaps Nova Scotia in the future) would benefit. I suspect they are likely to experience the same issues.*

**Response**: Yes, that is what we intended to mean, so we edited the sentence to be more clear.

**Line 48:** *The introduction is lengthy and should be revised to be more concise.*

**Response**: We have gone through and made some cuts, consolidated some paragraphs, and removed some of the unnecessary fish literature references to be much more focused on crustaceans

**Line 115-118** :add citation for this point or move one up from the following sentence

**Response**: Done. We edited the text a bit to be more general and included a reference. We also added a reference for the following line as it was missing one.

**Line 239**: *Add detail about why the hatchery crabs were used. Were they expected to have a different temperature tolerance due to different holding conditions? What were those conditions? Can a comment be added to the discussion that describes what the lack of a difference between wild and hatchery crabs from Chesapeake Bay means? Also, it would be helpful to add a sentence or two describing how crabs were held during transport from Chesapeake Bay and how long they were held under those conditions.*

**Response**: We used them because we needed a larger sample size for the CB group and know from previous studies that there are no differences in physiological endpoints (Johnson et al., 2011; Young et al., 2008). Survival and growth between hatchery reared and wild crabs released into the field are not different, and we added a new section the discussion (lines 704 – 718), where we address what the lack of difference between wild and hatchery might mean and highlight relevant literature on the subject matter. The holding conditions were identical between the NY crabs, hatchery crabs, and wild crabs from Chesapeake Bay once they were brought into the lab in NY. They were transported in nearly identical ways, but of course, we were very careful to regulate conditions in the CB crabs since the transport was longer. We added more details and information about both the holding conditions, transportation protocols, and hatchery practices to the methods section (lines 245 – 262). We confirmed that the hatchery collects a large number (80–120) of inseminated females from the wild every fall to serve as their broodstock in order to prevent inbreeding (Zohar et al., 2008). Therefore, the genetic diversity of hatchery crabs should be approximately the same as that of the crabs we attained from the wild.

**Line 310**: *It’s not entirely clear what data went into the different paragraphs of the results section. It seems like the first several paragraphs address the new data presented in this paper and the later paragraphs (e.g. starting line 390) include data from earlier studies. Clarifying which data are used in each part of the results would be very helpful to readers.*

**Response**: That is correct. I only bring in the previous data from other studies in the paragraph before the tables. I have edited the text to make that clear.

**Line 315**: *Calling all crabs >60 mm CW adults could be confusing if these were not all actually mature adult crabs. Perhaps a different name for this group would be better. This is comes up in the discussion line 596 when the authors have to distinguish mature adults.*

**Response**: We agree, and reviewer 1 also pointed this out. We decided to just use the terms small, medium, and large crabs throughout the text and in figures to avoid confusion.

**Line 456**: In general, the discussion is long and should be revised to be more concise.

**Response**: We have done some serious cutting and revision to the discussion. In particular, removed a lot of the fish literature references to focus just on crustaceans although some sections were added per suggestions from both reviewers.

**Line 460**: *Similar to the use of “bays” in the abstract, “origins” should be defined here or a different term should be used. Be consistent with these terms throughout.*

**Response**: We have changed it to estuary of origin throughout the text and in the figures.

**Line 496-490:** *This sentence is confusing. Rewrite to make the point clearer.*

**Response**: We edited that entire section to be more clear and concise.

**Line 534-551**: *Much of this paragraph is speculative and largely unrelated to the data presented in the paper. I recommend removing this paragraph and adding the most important points, such as the last sentence, to other sections. The emphasis on growth distracts from the more relevant points discussed in the paragraphs that follow.*

**Response**: We edited it significantly, added much more crab literature, removed the fish literature, and shortened it to discuss growth without distracting from the other points that follow.

**Line 599:** *Here or in the introduction it would be helpful to add detail about winter dredge fisheries. Do they occur in both places? Other parts of the range? Is this only a reference to the VA dredge fishery that has been closed on an annual basis for over a decade?*

**Response**: There was one short sentence about the winter dredge fisheries in the introduction, but we cut it. Yes, there are winter dredge fisheries in both bays (and probably in others as well), but we were intending to only refer to the winter dredge fishery in Great South Bay. We edited the text here a bit to make that distinction clear.

**Line 618:** *Many readers interested in the results may not know the term “pejus temperature” so using it only towards the end of the discussion could be confusing. I recommend using and defining the term in the introduction, probably in the paragraph starting with line 111.*

**Response**: Thank you for pointing this out. We changed it to “near the lower thermal limit”, which is more general.

**Line 624-636**: *The authors suggest that their models could be used to project the impacts of severe winters on populations. Is there a particular reason this was not done in this paper? The authors could make projections for Maryland waters of Chesapeake Bay and compare them to mortality estimates derived from Winter Dredge Survey field data. Even if there is no similar long-term set of field data for Great South Bay, conducting a similar model analysis and comparing the predicted mortality at this more northern site compared to Chesapeake Bay would be very interesting. For example, cold winters might cause 30% mortality in Chesapeake Bay and 60% in Great South Bay at a similar salinity.*

**Response**: We agree that it would have been very interesting. This is actually something we had wanted to do and even started doing, but for a variety of reasons it is not feasible or reasonable for this manuscript. To adequately model winter temperature in two large complex bays with unique circulation would have required more than what was in the scope of this paper. We thought about just calculating the average winter temperature at one site in each bay and then comparing the expected survival, but that seemed meaningless since this comparison would not account for the spatial heterogeneity of each estuary system. In fact, Bauer and Miller split these concepts into two separate manuscripts. The (Bauer & Miller, 2010a) paper is the experiment and modelling, while the (Bauer & Miller, 2010b) paper is the spatiotemporal application of the model. We will be applying this model to field data in another manuscript as part of a climate change population simulation study.

**Figure 6:** *Which data from the present study were included in the observed values. Were these Chesapeake Bay crabs, Great South Bay crabs, or both? Specify in the caption.*

**Response**: We can see how this might be confusing. For the observed values, we used all of the data from the present study including crabs from both bays. We edited the figure caption to be more specific about this.

We look forward to hearing from you in due time regarding our submission and to respond to any further questions and comments you may have.

Sincerely,

Adelle Molina

**References**

Bauer, L. J., & Miller, T. J. (2010a). Temperature-, salinity-, and size-dependent winter mortality of juvenile blue crabs (Callinectes sapidus). *Estuaries and Coasts, 33*(3), 668-677.

Bauer, L. J., & Miller, T. J. (2010b). Spatial and interannual variability in winter mortality of the blue crab (Callinectes sapidus) in the Chesapeake Bay. *Estuaries and Coasts, 33*(3), 678-687.

Brylawski, B. J., & Miller, T. J. (2003). Bioenergetic modeling of the blue crab (Callinectes sapidus) using the fish bioenergetics (3.0) computer program. *Bulletin of Marine Science, 72*(2), 491-504.

Johnson, E. G., Young, A. C., Hines, A. H., Kramer, M. A., Bademan, M., Goodison, M. R., & Aguilar, R. (2011). Field comparison of survival and growth of hatchery-reared versus wild blue crabs, Callinectes sapidus Rathbun. *Journal of Experimental Marine Biology and Ecology, 402*(1-2), 35-42.

Young, A. C., Johnson, E. G., Davis, J. L., Hines, A. H., Zmora, O., & Zohar, Y. (2008). Do hatchery-reared blue crabs differ from wild crabs, and does it matter? *Reviews in Fisheries Science, 16*(1-3), 254-261.

Zohar, Y., Hines, A. H., Zmora, O., Johnson, E. G., Lipcius, R. N., Seitz, R. D., . . . Stubblefield, J. D. (2008). The Chesapeake Bay blue crab (Callinectes sapidus): A multidisciplinary approach to responsible stock replenishment. *Reviews in Fisheries Science, 16*(1-3), 24-34.
