# Supplementary material for: Population level differences in overwintering survivorship of blue crabs (Callinectes sapidus): A caution on extrapolating climate sensitivities along latitudinal gradients
Source: PLoS One. 2021 Sep 21;16(9):e0257569. doi: 10.1371/journal.pone.0257569 (PMC8454986; doi:10.1371/journal.pone.0257569)
Supplement: Attachment — Submitted filename: Response to Reviewers.docx [file pone.0257569.s006.docx]

Dear Mr. Charles William Martin,

Thank you for giving me the opportunity to submit minor revisions to the draft of my manuscript titled “Population level differences in overwintering survivorship of blue crabs (Callinectes sapidus): a caution on extrapolating climate sensitivities along latitudinal gradients” to *PLOS ONE*. We appreciate the time and effort that you and the reviewers have dedicated to providing your valuable feedback on my revised manuscript. We are very grateful that both reviewers believe we have addressed their major concerns and are happy to incorporate the minor revisions that have been suggested.

Here is a point-by-point response to the reviewers’ comments.

**Journal Requirements**

Please review your reference list to ensure that it is complete and correct. If you have cited papers that have been retracted, please include the rationale for doing so in the manuscript text, or remove these references and replace them with relevant current references. Any changes to the reference list should be mentioned in the rebuttal letter that accompanies your revised manuscript. If you need to cite a retracted article, indicate the article’s retracted status in the References list and also include a citation and full reference for the retraction notice.

We have reviewed the reference list and are certain that it is both complete and correct. We have not cited any retracted papers, to our knowledge. We have not made any changes to the reference list in this final revision.

**Reviewer 1**

The authors addressed all of my previous comments, and I believe the manuscript is much improved. A few minor editorial comments, by line number:

130: there is a space before the comma but not after.

I decided to turn this into two sentences. Deleted the comma and the word but then added a period a space and capitalized the first word of the next sentence (Recent)

140: there is an extra space and extra period after the period.

Deleted the extra space and the period.

131 and 321: I suggest writing p < 0.001 instead of p = 4e-04 or similar.

I agree. I had used this syntax later in the paragraph. I have edited all of them to use the same format.

377 and elsewhere mentioning interactions: use a multiplication symbol rather than an x.

Changed this in the text as well as in the tables and supplementary tables.

**Reviewer 2**

The manuscript “Population level differences in overwintering survivorship of blue crabs (Callinectes sapidus): a caution on extrapolating climate sensitivities along latitudinal gradients” describes laboratory studies and modeling to explore the mortality of blue crabs across a range of temperatures, salinities, and sizes. The authors have effectively addressed the comments of the reviewers and I only noted a number of minor corrections that should be made before the manuscript is published. These minor corrections are listed below.

L53 – I think there is a comma missing after the word “growth”

Yes thank you I added a comma there to close out the such as clause.

L71 – changing “to an entire species” to “to a species across its entire range” might make more sense here

I agree; I changed it.

L79 – Period missing after references 22-23

Corrected

L93 – I think the point you are making here would be clearer if you changed this to “quantify underlying variation which can…”

That does sound much better

L129-133 – this is a long sentence. Splitting it into two sentence it might help the reader understand the points you make here.

I did so

L109-112 – fix punctuation in this sentence

I added a comma between salinity and size and another one after temperature to close out that clause.

L118 – I think “Blue crabs are less tolerant” would be better here

I agree. Past tense was weird.

L164 – missing punctuation

Yes, I added a space between period and while.

L280 – missing reference

If you are referring to the () after flexsurvreg, that is to denote a function in R, not a missing reference.

L431 – delete comma after “salinity”

Done

L440 – there’s something wrong here “of temperature of salinities” doesn’t make sense

That was a typo. Deleted of and changed to and

L460 – missing period

Added a period after size.

L527-530 – This is an interesting point about increased salinity provide a refuge. Another result of higher survival at higher salinities might be that high salinity water is a spatial refuge for at least some individuals of all sexes and sizes. If you were going to track the northward range expansion of blue crabs, it seems you would need to focus sampling on the saltier habitats. This might be an interesting point to add to the discussion but is only a minor suggestion.

I added the following sentence at line 522-523 “It may also signify that high salinity can provide a spatial refuge in winter.” to discuss this idea because I think it is important and something I missed touching on.

L543-544 – redundant use of “in the Chesapeake”

Removed the Chesapeake from the intro clause but left it at the end.

L631 – the term “good” is very vague here. Define this more specifically. It may be good for crabs and crab fishermen but bad for crab prey and fishermen targeting other species like perhaps lobster.

I agree. I changed it from good to “beneficial for blue crab population growth rates” but removed “on blue crab populations” from later in the sentence because it is repetitive.

**Other Changes**

Upon reviewing the manuscript one last time, we also made the following minor changes. Most of these were formatting, such as missing or extra spaces and/or punctuation, primarily from track changes.

Table 1: Removed units from the column labels in parentheses and added it to the caption

Line 78: Added space before the reference

Line 87: Added a space before the reference

Line 110: Added a space between but and have

Line 113: Deleted extra space after the period

Line 246: Removed extra space after both

Line 306: Added a space after comma

Line 307: Changed recruits, subadults, and adults to small, medium, and large respectively

Line 392: Added a space after from, before the reference

Line 431: Removed the comma after salinity

Line 431: Removed the comma after salinity

Line 434: Added a comma after salinity

Line 490: Removed a space after because

Line 501: Removed extra space after the period

Line 510: Removed extra space and replaced comma with a period

Line 519, 520, 534, 536, 540, 557, 586, 627, 629, 632, 635: Removed extra space after the periods

Line 548 & 594: Removed extra space after the

We look forward to hearing from you again regarding our submission and are happy to respond to any further questions and comments you may have.

Sincerely,

Adelle Molina
